# Synthesized natural peptides from amphibian skin secretions increase the efficacy of a therapeutic vaccine by recruiting more T cells to the tumour site

**DOI:** 10.1186/s12906-019-2571-z

**Published:** 2019-07-06

**Authors:** Xuan Pan, Bowei Ma, Xinchao You, Shu Chen, Jialing Wu, Tianfang Wang, Shelley F. Walton, Jianwei Yuan, Xiaolian Wu, Guoqiang Chen, Yuejian Wang, Guoying Ni, Xiaosong Liu

**Affiliations:** 1 The First Affiliated Hospital/Clinical Medical School Department of Nuclear Medicine, Guangdong Pharmaceutical University, Nong Lin Xia Lu, Yuexiu District, Guangzhou, 510080 Guangdong China; 2 Cancer Research Institute, Foshan First People’s Hospital, Foshan, 528000 Guangdong China; 3 Inflammation and Healing Research Cluster School of Health and Sport Sciences, University of Sunshine Coast, QLD, Maroochydore DC, 4558 Australia; 4 Genecology Research Centre, University of Sunshine Coast, QLD, Maroochydore DC, 4558 Australia

**Keywords:** Caerin peptide, Therapeutic vaccine, Cervical cancer, Tumour microenvironment

## Abstract

**Background:**

Therapeutic vaccines against cervical cancer remain ineffective. Previously, we demonstrated that blocking the signalling of a cytokine, interleukin 10, at the time of immunisation elicited significantly higher numbers of antigen specific T cells and inhibited tumour growth in mice.

**Results:**

In the current paper, we demonstrate, in a HPV16 E6/E7 transformed TC-1 tumour mouse model, that despite increased antigen specific T cell numbers, blocking IL-10 signalling at the time of immunisation does not increase the survival time of the TC-1 tumour bearing mice compared to mice receiving the same immunisation with no IL-10 signalling blockade. Moreover, the function of tumour infiltrating T cells isolated 3 weeks post TC-1 transplantation is more suppressed than those isolated 2 weeks after tumour inoculation. We demonstrate that synthesized caerin peptides, derived from amphibian skin secretions, 1) were able to inhibit TC-1 tumour growth both in vitro and in vivo; 2) are environmentally stable; and 3) promote the secretion of pro-inflammatory interlukine-6 by TC-1 cells. Notably caerin peptides were able to increase the survival time of TC-1 tumour bearing mice after therapeutic vaccination with a HPV16E7 peptide-based vaccine containing IL-10 inhibitor, via recruiting increased levels of T cells to the tumour site.

**Conclusion:**

Caerin peptides increase the efficacy of a therapeutic vaccine by recruiting more T cells to the tumour site.

## Background

Cervical cancer is the 2nd most common cancer in woman worldwide, and accounts for around 250,000 deaths each year, especially in developing countries [1, 2]. Cervical cancer results from persistent high-risk human papillomavirus (HPV) infection, mostly HPV subtypes 16 and 18, which are responsible for around 70% of cervical cancers. A prophylactic vaccine, based on the papillomavirus like particles (VLPs), has been available to the public since 2006, but the vaccine is not effective for those already infected [3–6]. During the past few years, a better understanding of the HPV-host immune system interaction and the development of new therapeutics targeting immune check points have renewed interest in the use of immunotherapy for cervical cancer treatment.

Therapeutic vaccines only target tumour cells but leave normal tissues and organs unaffected. Ideally, therapeutic tumour vaccines should produce sufficient high-quality effector CD8+ T cells that can migrate to the tumour site, overcome the tumour immune suppressive environment and kill the tumour cells [7]. Peptide based therapeutic vaccines are easier to produce with less side effects, and therefore have been studied extensively [8]. In one such study, women with HPV-16-positive, grade 3 vulvar intraepithelial neoplasia were vaccinated with a mix of long peptides from the HPV16 E6 and E7 in incomplete Freund’s adjuvant. Fifteen of 19 patients had a clinical response, with complete viral clearance observed in 9 of 19 patients [9]. Vaccine induced memory CD8+ T cell responses predict the therapeutic efficacy of a therapeutic vaccine [10]. The same vaccine was also able to induce a broad IFN*γ*-associated T-cell response in patients with advanced or recurrent HPV16-induced gynaecological carcinoma but did not induce tumour regression or prevent progressive disease [8]. Therefore, the efficacy of the therapeutic vaccine against cervical cancer remains to be improved.

Interleukin 10 (IL-10) is a cytokine with multiple biological function [11]. The main function of IL-10 is to limit immune responses against foreign and self-antigens, to avoid excessive immune response damage to self, via inhibiting the function of professional antigen presenting cells [12]. Temporal blockade of IL-10 at the time of immunization drastically increases vaccine-induced CD8+ T cell responses [13, 14]. This phenomenon can be observed when HPV VLPs, soluble antigen, peptide, or DNA are used as immunogens [14]. Compared with vaccination using HPV16 long E7 peptide in incomplete Freund’s adjuvant on its own, blocking IL-10 at the time of immunisation elicits significantly higher numbers of CD8+ T cells and attracts more CD4+ and CD8+ T cells to the tumour site [15, 16]. However, the survival time of HPV16 E6/E7 transformed TC-1 tumour bearing mice is similar between the two immunisation groups. Therefore, the tumour microenvironment is critical and might determine the efficacy of a therapeutic vaccine.

The tumour microenvironment consists of tumour cells, immune suppressive cells such as T Regulatory T cells, tumour associated macrophages, myeloid derived immune suppressive cells, and cytokines with immune suppressive function [17, 18]. Although the presence of T cells within the tumour indicates a better prognosis for the cancer patient, tumour infiltrating T cells are usually anergic, secreting less cytokines and responding poorly to specific and non-specific stimulation [19]. Attempts to overcome the tumour microenvironment have been extensively studied, include targeting the PD-L1–PD-1 axis, Indoleamine-pyrrole 2, 3-dioxygenase (IDO), T regulatory cells, and T cell–intrinsic energy [19]. Monoclonal antibodies targeting PD-1/PD-L1 axis shows efficacy in about one third of melanoma patients. Another example is the use of anaerobic bacteria, which only proliferate in the anoxic environment within the centre of a tumour [20]. Toll like receptor ligands, such as CpG and imiquimod, have also been used to break the immune suppressive microenvironment [21].

Peptides isolated from amphibians have been shown to lyse bacteria via a unique mechanism possibly involving cell membrane lysis. Some of these peptides are observed to be highly active against cancer cells but not normal mammalian cell. More than 200 host-defence peptides have been isolated and identified from skin secretions of Australian frogs and toads. Many of these peptides have antimicrobial and/or neuropeptide-type activities (27–29). The caerin 1 peptides have previously been shown to be potent membrane-active peptides and to stop the formation of nitric oxide by neuronal nitric oxide synthase. Caerin 1.1 (^1^GLLSVLGSV^10^AKHVLPHVLP^20^HVVPVIAEHL-NH_2_) has an anti-cancer effect against several human cancer cell lines. The caerin 1.9 peptide (^1^GLFGVLGSI^10^AKHVLPHVVP^20^VIAEKL-NH_2_) has antimicrobial activity against a wide spectrum of Gram-positive and Gram-negative microbial strains. Caerin 1.1 and 1.9 are originally isolated from skin secretions of Australian tree frog *Litoria splendida.* Caerin 1.1 and 1.9 inhibit HIV-infected T cells within minutes post-exposure at concentrations non-toxic to T cells and inhibit the transfer of HIV from dendritic cells (DCs) to T cells [22]. We have recently shown that caerin 1.1 and 1.9 have cytotoxicity to HPV 16 early protein E6/E7 transformed TC-1 cells in vitro, and the anti-cancer effects were more profound when caerin 1.1 and 1.9 were used together [20]. Moreover, proteomics analysis showed that caerin 1.9 could stimulate multiple signalling pathways including several pro-inflammatory signalling pathways [20, 23, 24].

In the current paper, we investigated the function of tumour infiltrating T cells at various stages after tumour transplantation in a TC-1 tumour mouse model and showed that caerin 1.1 and 1.9 were able to stimulate the secretion of IL-6 by TC-1 cells. Moreover, caerin 1.1 and 1.9 increased the efficacy of a therapeutic vaccine containing IL-10 inhibitor in the TC-1 mice tumour model by increasing the survival time of TC-1 tumour bearing mice.

## Methods

### Mice

Six to eight weeks old, specific pathogen free (SPF) adult female C57BL/6 (H-2b) mice and Nude mice were ordered from the Animal Resource Centre, Sun Yat-Sen University and kept at the Animal Resource Centre, the first affiliated hospital of Guangdong Pharmaceutical University, Guangdong province, China. Experiments were approved by and then performed in compliance with the guidelines of Animal Experimentation Ethics Committee (Ethics Approval Number: FAHGPU20160316) of the aforementioned hospital.

All mice were kept at SPF condition on a 12-h light/12-h dark cycle. The temperature of the animal house was 22 °C and the humidity was 75%. 5 mice were kept each cage, provided with sterilised standard mouse food and water. TC-1 tumour bearing mice were given 1% sodium pentobarbital by *i.p.* injection when treatment was performed. Mice were sacrificed by CO_2_ inhalation at the end of each experiment and confirmed by the ceasing of heart beat.

### Cell line, peptide synthesis and antibodies

A murine TC-1 cell line transformed with HPV16 E6/E7 was obtained from Shanghai Institute for Cell Resources Centre, Chinese Academy of Sciences, and cultured following the protocols in the product sheets. The culture of TC-1 cells was described elsewhere [16]. Briefly, TC-1 cells were cultured at 37 °C with 5% CO_2_ in complete RPMI 1640 media (GIBCO) supplemented with 10% heat inactivated fetal calf serum (FCS, GIBCO), 100 U of penicillin/mL and 100 mg of streptomycin/mL (GIBCO), 0.2 mM non-essential amino acid solution, 1.0 mM sodium pyruvate, 2 mM L-glutamine, 0.4 mg/mL G418.

Human cervical cancer cell Hela and stable cell line of T-SV4O immortalized human glomerular mesangial cell (HMC) were purchased from the Shanghai Institute for Biological Sciences, Chinese Academy of Sciences. The cell lines were grown in RPMI 1640 media (GIBCO) supplemented with 10% heat inactivated fatal calf serum (FCS, GIBCO), 100 U of penicillin/mL and 100 mg of streptomycin/mL (GIBCO), in humidified atmosphere of 5% CO_2_ at 37 °C.

Caerin 1.1 and Caerin 1.9 peptides from Australian tree frog *Litoria splendida* are synthesized and therefore does not include any actual materials from frogs.

Caerin 1.1 (GLLSVLGSVAKHVLPHVLPHVVPVIAEHL-NH_2_) and caerin 1.9 (GLFGVLGSIAKHVLPHVVPVIAEKL-NH_2_), control peptide (GTELPSPPSVWFEAEFK), HPV16 E7 CTL epitope RAHYNIVTF, and four overlapping peptides representing the entire HPV 16 E7 protein, EX (MHGDTPTLHEYMLDLQPETTDLYCYEQLNDSSEEE, LNDSSEEEDEIDGPAGQAEPDRAHYNIVTFCCKC, DRAHYNIVTFCCKCDSTLRLCVQSTHVDIR, CVQSTHVDIRTLEDLLMGTLGIVCPICSQKP were synthesised by Mimotopes Proprietary Limited, Wuxi, China. The purity of the peptides was > 95% as determined by reverse-phase HPLC at Mimotopes. The lipopolysaccharide concentration of F1, F3 and P3 was 0.03EU/ml, 0.03EU/ml and 0.44EU/ml respectively as measured by Kinetic Turbidimetric Assay by Xiamen Bioendo Technology Co., Ltd.

For immunisation, anti-IL10 receptor (1B1.3) monoclonal antibody (MAb) and IgG Isotype control antibody (LTF-2) were purchased from BioXcell, USA and stored at − 80 °C till further use. Anti-Mouse CD3 FITC (17A2) was purchased from BioLegend (San Diego USA). Anti-Mouse IFN-*γ* PE (XMG1.2), anti-Mouse Granzyme B PE (NG2B), anti-Mouse Perforin PE (eBio0MAK-D), anti-Mouse CD4 PerCP-Cyanine5.5 (RM4–5), anti-Mouse CD8 PerCP-Cyanine5.5 (53–6.7) and anti-Mouse CD279 (PD-1) APC (J43) were purchased from eBioscience (Waltham, USA).

### Intracellular staining

Tumours were excised and cut into small pieces after removal of blood vessels and connective tissue. To isolate blood cells, tumour tissue was incubated for 1 h, with occasional shaking, in an enzyme mixture that consisted of 1 mg /ml of collagenase D, 20 mg /ml of DNase I (Roche), and 10% fetal calf serum in RPMI-1640 at 37 °C. The digested tissue was filtered through a 70 μm nylon mesh, and the resultant cells were washed twice in PBS. Mononuclear cells were obtained with Lymphocyte Separation Medium (Sigma-Aldrich) following centrifugation at 2000 rpm for 25 min. The intra-cellular staining has been described elsewhere [15]. Briefly, mononuclear cells were harvested and incubated with cell stimulation cocktail (eBioscience) in the presence of 2 μM of monensin (eBioscience) at a density of 1 × 10^6^ cells/ml overnight at 37 °C with 5% CO_2_. Cells were stained with antibody against CD3, CD4 and CD8 and then fixed and permeabilized using permeabilization buffer (Biolegend) before intra-cellularly stained with anti-mouse IFN-*γ*, anti-Mouse Granzyme B, anti-Mouse Perforin or isotype-matched control mAb for 20 min in the dark at room temperature. Samples were analysed by flow cytometry using a FACS Calibur analyser (Becton Dickinson).

### MTT assay

Cell proliferation was determined by MTT assay (ATCC, USA) following the manufacturer instructions as described elsewhere [20]. Briefly, 5 × 10^3^ of TC-1 cells were cultured in flat bottomed 96 well plates before adding approximately 0–15 μg of peptides was added to 5 × 10^3^ TC-1, Hela, or MHC cells and cultured overnight at 37 °C with 5% CO_2_, followed by adding ten microliters of MTT stock solution and cultured another 4 h. 100 μl of DMSO was added to stop the experiment. Each treatment was performed in triplicate. Results were analysed by an ELISA plate reader (BioTek, USA) at 570 nm according to the manufacturer’s protocol.

### Cytokine ELISA

Cytokine ELISA kits for the detection of IL-1α and MCP-1, IL-10 and IL-6 were purchased from eBioscience. IL-1α, IL-6, IL-10 and MCP-1 were detected from the supernatants of caerin peptide or as a control imiquimod treated TC-1 cells following the protocols provided by the manufacturer.

### Tumour challenge

TC-1 tumour challenge has been described elsewhere (41). Briefly, TC-1 cells, at approximately 70% confluency, were harvested with 0.25% trypsin and washed repeatedly with PBS. TC-1 tumour cells (3 × 10^5^ /mouse) in 0.1 ml of PBS were injected subcutaneously into the left flank. Tumour sizes were assessed every 3 days using callipers to determine the average diameter of each tumour. Tumour volumes were calculated as width×width×length. Mice were sacrificed when the tumour diameter reached 20 mm. In another experiment, TC-1 tumour bearing mice were sacrificed and the tumour dissected and weighted using an electronic scale (Sartorius, Germany).

### Immunization of mice

Four to five days post TC-1-challenge, groups of five to eight mice were immunised subcutaneously (*s.c.*) with vaccine containing either 40 mg of four overlapping HPV16E7 peptides (EX) (10 μg/each), 15 mg of monophosphoryl lipid A (MPLA) (Sigma-Aldrich), with or without 250 mg of anti-IL10R antibodies, or IgG Isotype Control Antibody (LTF-2), dissolved in PBS. The total injected volume was 100 μL/mouse.

### Tumour local administration of caerin peptides

Four to five days post-TC-1-challenge, when the tumours’ diameters reached 3 to 5 mm, the mice either immunised or unimmunised were intratumorally injected with caerin peptides (Caerin 1.1 and Caerin 1.9), or PBS for six consecutive days.

### ELISPOT

ELISPOT was performed as described previously (37). Briefly, single spleen cell suspensions were added to membrane base 96 well ELISPOT plate (Millipore, Bedford, MA) coated with anti-IFN-*γ* (BD Harlingen, San Diego, CA). HPV16 E7 CTL epitope RAHYNIVTF was added at various concentrations and cells incubated with the peptide overnight at 37 °C with 5% CO_2_. The plate was incubated with detection antibody (a biotinylated anti-mouse IFN-*γ* antibody, BD Harlingen, San Diego, CA) after extensive washing in wash buffer for 2 h at room temperature. Antigen specific IFN-*γ* secreting cells were detected by sequential exposure of the plate to avidin–horseradish peroxidase (Sigma-Aldrich) and DAB (Sigma-Aldrich). The plate was washed, allowed to air dry overnight, foci of staining were counted by ELISPOT reader system (CTL, Germany).

### Statistical analysis

Data are expressed as the mean ± SD and statistical significance was determined using paired two-tailed Student t test. Survival rate’s comparison between different groups was performed by log rank test, by using Prism 5.0 (Graphpad Software, San Diego). Results were considered significant if *P* value was less than 0.05.

## Results

### Blocking IL-10 at the time of immunisation does not increase the survival time of TC-1 tumour bearing mice

**Fig. 1:**
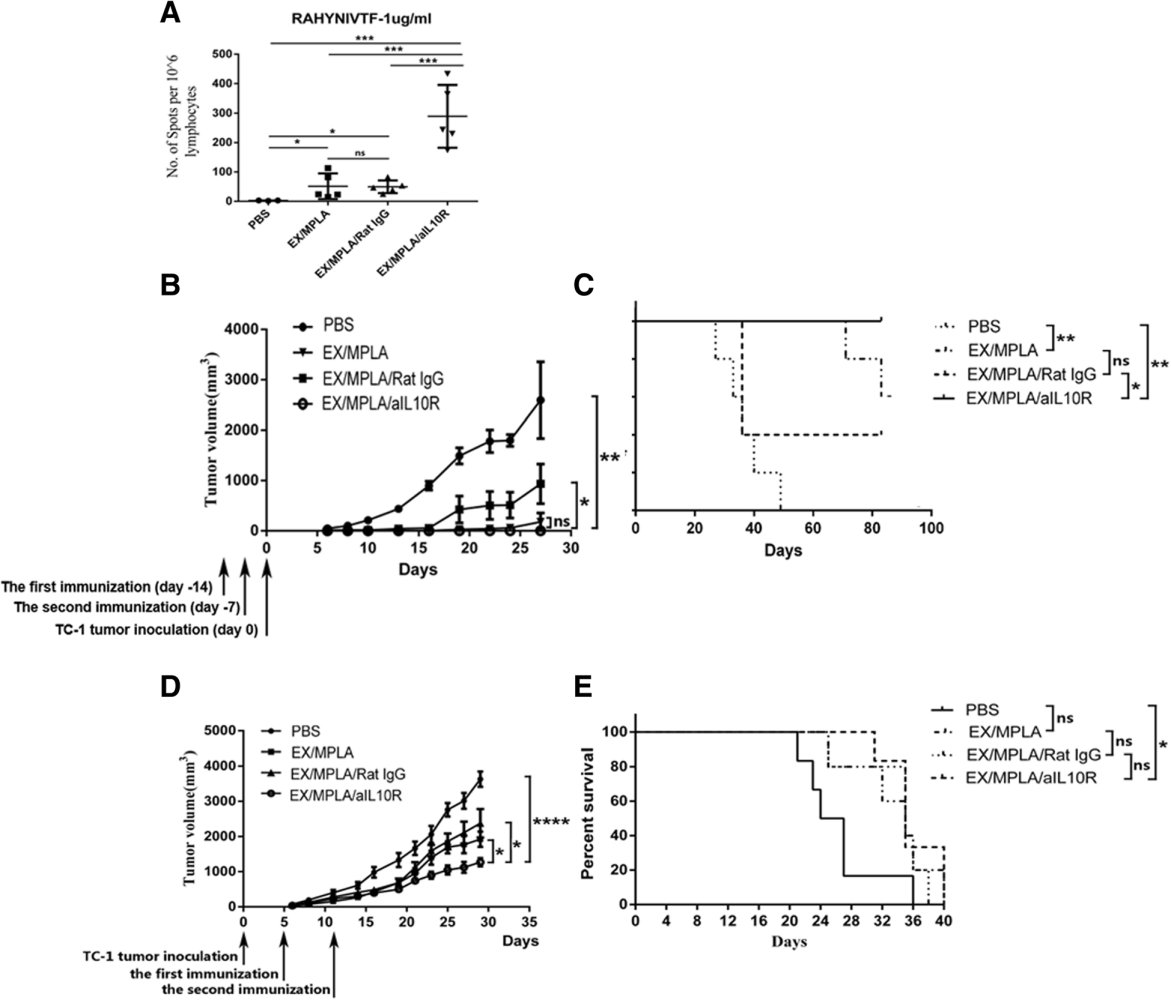
Blocking IL-10 at the time of immunisation does not increase the survival time of TC-1 tumour bearing mice **a** Groups of five C57BL/6 mice were primed and boosted on day 0 and day 7 respectively with 40 μg of Ex peptides (each 10 μg), 15 mg of MPLA and 250 μg of anti-IL10 receptor antibodies or IgG Isotype control antibody subcutaneously. Seven days after final immunisation, spleens from immunized mice were collected, single spleen cells isolated and cultured in the presence of an MHC I restricted HPV16 E7 specific peptide RAHYNIVTF. ELISPOT assay for IFN*γ* was performed as described in Materials and Methods. **b, c** Groups of five C57BL/6 mice were immunized with 40 μg of Ex peptide, 15 mg of MPLA and 250 μg of anti-IL10 receptor antibodies or IgG isotype control antibody subcutaneously or left unimmunized on day zero and seven. Seven days after immunization, 2 × 10^5^ of TC-1 tumour cells were inoculated subcutaneously in the flank of immunized or unimmunised mice, and tumour size and survival of mice were monitored as described in Materials and Methods. (D, E) Groups of eight C57BL/6 mice were inoculated subcutaneously in the flank with 2 × 10^5^ of TC-1 tumour cells. 5 days later, mice were immunized with 40 mg of Ex peptide, 15 mg of MPLA and 250 μg of anti-IL10 receptor antibodies or IgG isotype control antibody subcutaneously or left unimmunized twice 7 days apart. Survival of mice was monitored as described in Materials and Methods. The results represent at least one of two independent experiments.

Previous studies show temporal blocking IL-10 at the time of immunisation drastically increases vaccine induced antigen specific CD8+ T cell numbers [14, 25]. We wish to investigate whether blocking IL-10 at the time of immunisation, which increases the numbers of antigen specific IFNγ CD8+ T cells, better prevent TC-1 tumour growth in mice than immunisation without IL-10 signalling blockage, especially in therapeutic setting. C57/BL6 mice were immunised twice, 7 days apart, with four overlapping peptides representing the entire sequence of HPV16 E7 protein (EX)/MPLA with or without simultaneous administration of anti-IL-10R antibodies. Control groups including PBS mock immunised and EX/MPLA/control antibody immunised group. As expected, blocking IL-10 significantly increased E7 specific IFN*γ* secreting CD8+ T cell numbers compared with mice immunised without IL-10 signalling blockade measured by ELISPOT (Fig. 1a).

Next, C57/BL6 mice were immunised with EX/MPLA twice with an interval of 7 days, with or without IL-10 signalling blockade. Control groups included unimmunised and EX/MPLA immunisation plus a control antibody. Seven days after final immunisation, the mice were challenged subcutaneously with TC-1 tumour cells. Mice that received EX/MPLA and IL-10R antibody immunisation did not have any observable tumour growth, while tumour growth was observed in all other groups after tumour challenge. Moreover, 100% of mice immunised with EX/MPLA together with IL-10R antibody survived 80 days post tumour challenge (Fig. 1b and c). Between 40 and 60% of mice in EX/MPLA only and EX/MPLA control antibody groups survived over 80 days. The survival time was observed to be similar between the EX/MPLA/control antibody and the EX/MPLA only immunisation group 80 days post TC-1 tumour inoculation. All mice from untreated group were dead within 50 days after tumour challenge.

Following this, C57BL/6 mice were subcutaneously inoculated with TC-1 tumour cells. Five days after tumour challenge, TC-1 tumour bearing mice were immunised with EX/MPLA with or without IL-10 signalling blockade, controls include EX/MPLA/control antibody and unimmunised groups. Thirty days after TC-1 tumour inoculation, mice immunised with the EX/MPLA and IL-10 blockade demonstrated increased inhibition of TC-1 tumour growth compared to mice in groups immunised without the IL-10 signalling blockade or with control antibody. However, the survival time among the immunisation groups were similar, whether the tumour bearing mice were immunised with or without the IL-10 signalling blockade (Fig. 1d and e). The survival time of mice immunised with EX/MPLA and anti-IL-10R antibody was statistically longer than that of unimmunised control (*P* < 0.05).

### Tumour infiltrating T cells, isolated 3 weeks were more suppressed compared with those isolated 2 weeks after tumour inoculation

**Fig. 2:**
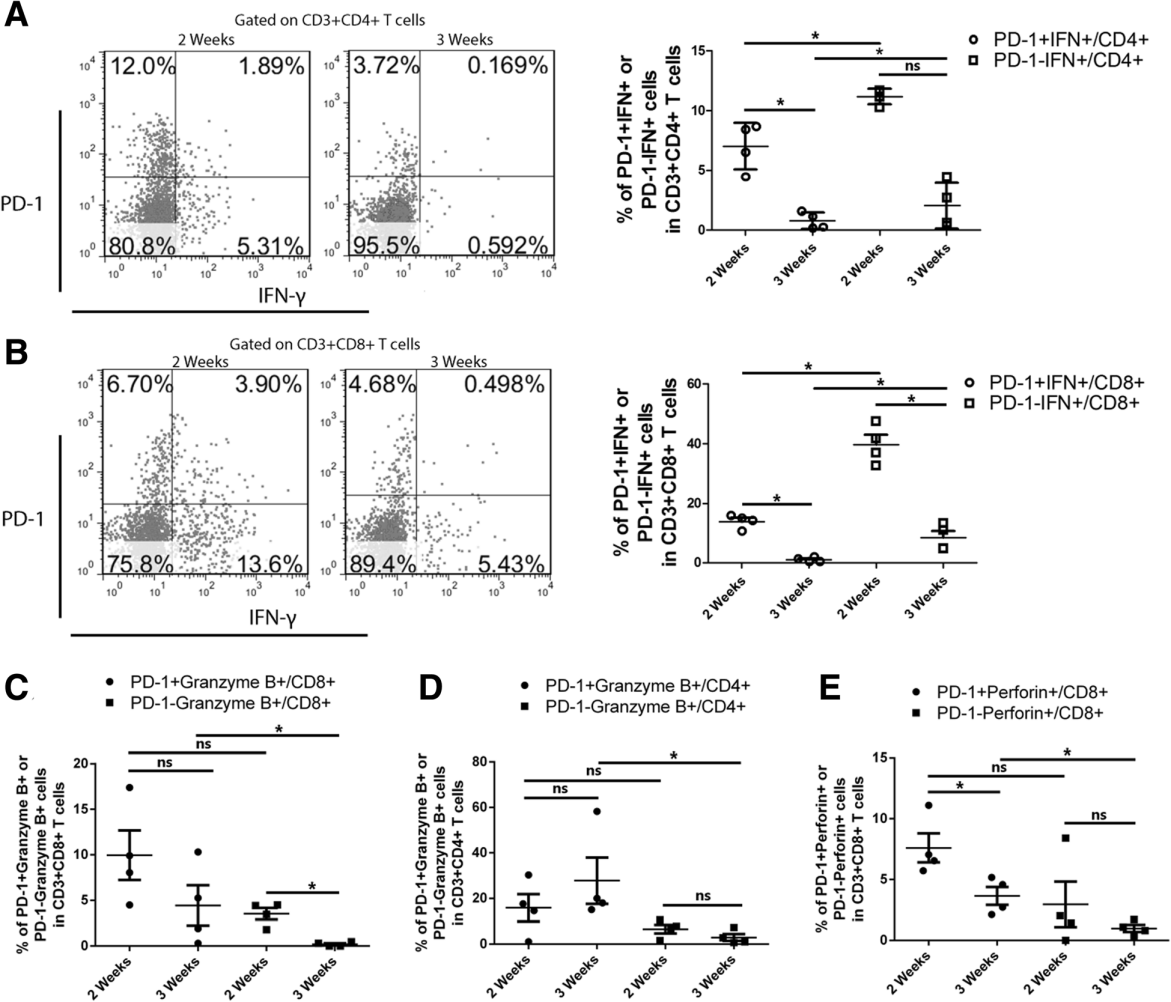
Tumour infiltrating T cells isolated three weeks after transplantation are more inhibited Groups of four mice were subcutaneously inoculated with 2 × 10^5^ of TC-1 tumour cells in the flank. Mice were terminated at 2 or 3 weeks after TC-1 tumour inoculation. Tumours were dissociated and digested with collagenous D and DNase I. Mononuclear cells were harvested by Ficoll separation and incubated with cell stimulation cocktail in the presence of monensin overnight at 37 °C/5% CO_2_. Cells were surface stained for CD3, CD4 or CD8, and PD-1 followed by intracellular staining for IFN-*γ*, granzyme B or perforin. (**a)** Percentage PD-1 + IFN-*γ* + or PD-1-IFN-*γ* + of CD3 + CD4+ cells; (**b)** Percentage of PD-1 + IFN-*γ* + or PD-1-IFN-*γ* + of CD3 + CD8+ cells; (**c**) Percentage of PD-1+ granzyme B+ or PD-1- granzyme B+ of CD3 + CD8+ cells; (d) Percentage of PD-1+ granzyme B+ or PD-1- granzyme B+ of CD3 + CD4+ cells; (**e**) Percentage of PD-1+ perforin+ or PD-1- perforin+ of CD3 + CD8+ cells. The results represent one of two independent experiments.

We then investigated the function of tumour infiltrating T cells isolated from mice bearing TC-1 tumour. Two or three weeks after subcutaneous TC-1 tumour inoculation, tumour infiltrating T cells were isolated and stained for T cell surface markers followed by intracellular staining of cytokines and molecules related to their tumour killing function. Both CD4+ and CD8+ T cells isolated 3 weeks post inoculation secreted less IFN*γ* than CD4+ and CD8+ T cells isolated 2 weeks after tumour inoculation, whether T cells were PD-1+ or PD-1- (Fig. 2a and b). CD8+ T cells isolated 3 weeks after TC-1 inoculation also secreted less Granzyme B and Perforin compared with those isolated 2 weeks after inoculation, although those were not as statistically significant as IFN*γ* (Fig. 2c and e). CD4+ T cells secreted similar levels of granzyme B, whether isolated two or three weeks after TC-1 transplantation (Fig. 2d). These results suggest that the tumour microenvironment severely hampers the function of infiltrating T cells once established. Disturbing the tumour microenvironment has potential to increase the efficacy of a therapeutic vaccine.

### Caerin peptides are stable

**Fig. 3:**
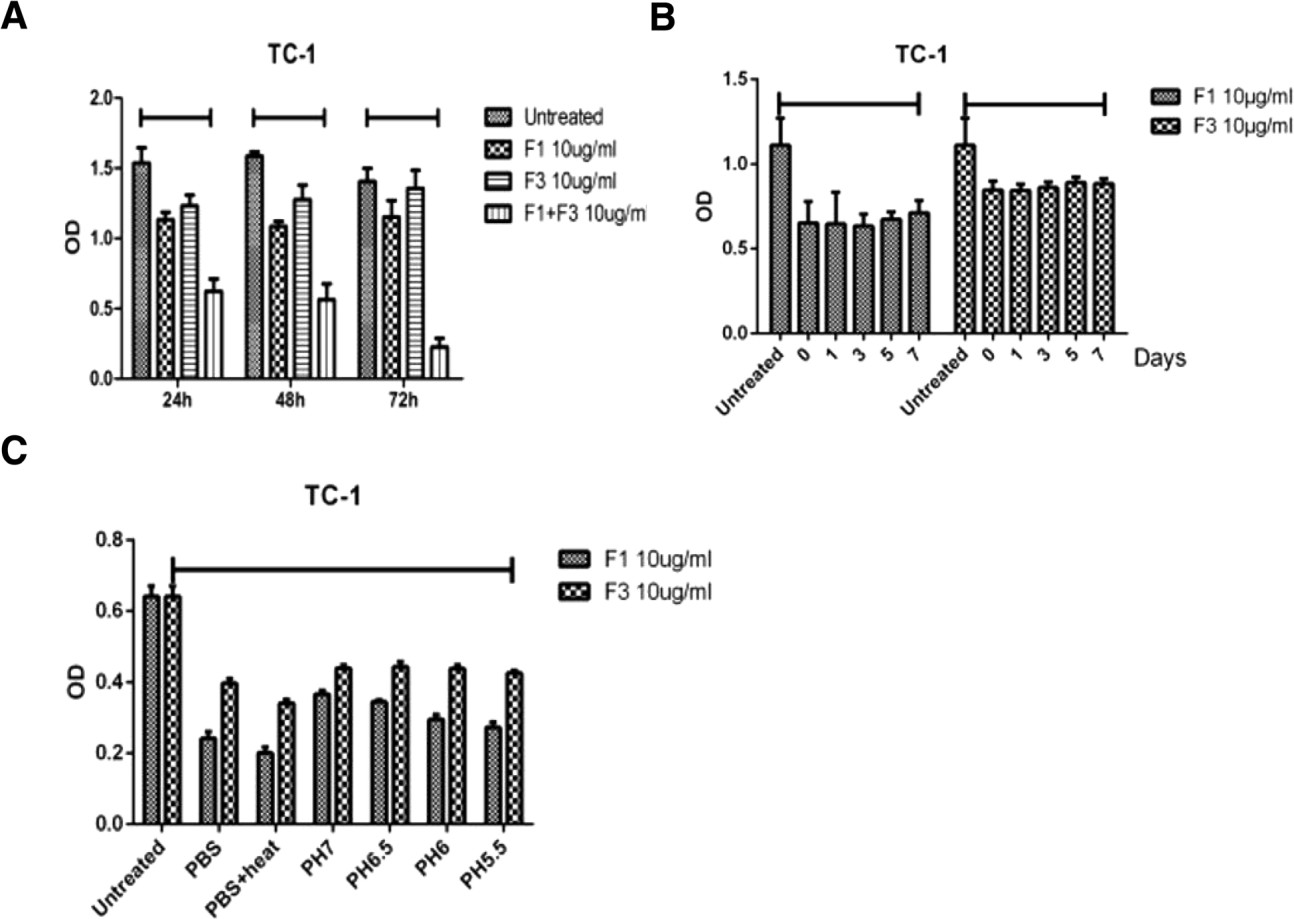
Caerin peptides are stable 5 or 10 μg of Caerin 1.1 (F1) and Caerin 1.9 (F3) peptides were added to 8 × 10^3^ of TC-1 cells in flat bottomed 96 well plates and cultured overnight at 37 °C with 5% CO_2_. Cell proliferation was determined by an MTT assay. (**a**): MTT assay after TC-1 cells were incubated with Caerin 1.1 for 24–72 h. (**b**): Caerin 1.1 and 1.9 were left at room temperature for up to 7 days before processing to an overnight MTT assay. (**c**): MTT assay after the pH of the PBS was adjusted to acidic condition (from pH 7.4 to pH 5.5), and after the Caerin 1.1 and 1.9 was heated at 100 °C for 15 min. *:*P* < 0.05; **: *P* < 0.01; ***: *P* < 0.001.

Recently, we demonstrated that caerin peptides isolated from the skin secretions of Australian tree frog were able to inhibit the viability of TC-1 cell growth in vitro [20]. Here we demonstrate the stability of the caerin peptides to disturb the tumour microenvironment. First, a three-day MTT assay was employed to investigate whether caerin peptides were able to inhibit the viability of TC-1 cell growth (Fig. 3a). After 72 h caerin 1.1 and 1.9 were able to inhibit the viability of TC-1 cell growth at 37 °C. Next, caerin 1.1 and 1.9 were placed at room temperature for 7 days before being used in an MTT assay, results showed a similar level of inhibition compared to those stored at -20 °C prior to the assay (Fig. 3b). Caerin 1.1 and 1.9 were also able to inhibit TC-1 cell growth in vitro, with 50–60% of cell growth inhibition respectively when the pH of PBS used to dissolve the peptides was adjusted to acidic condition (from pH 7.4 to pH 5.5), and after caerin 1.1 and 1.9 was heated at 100 °C for 15 min (Fig. 3b and c).

### Caerin 1.1 and 1.9 inhibit TC-1 growth in vivo

**Fig. 4:**
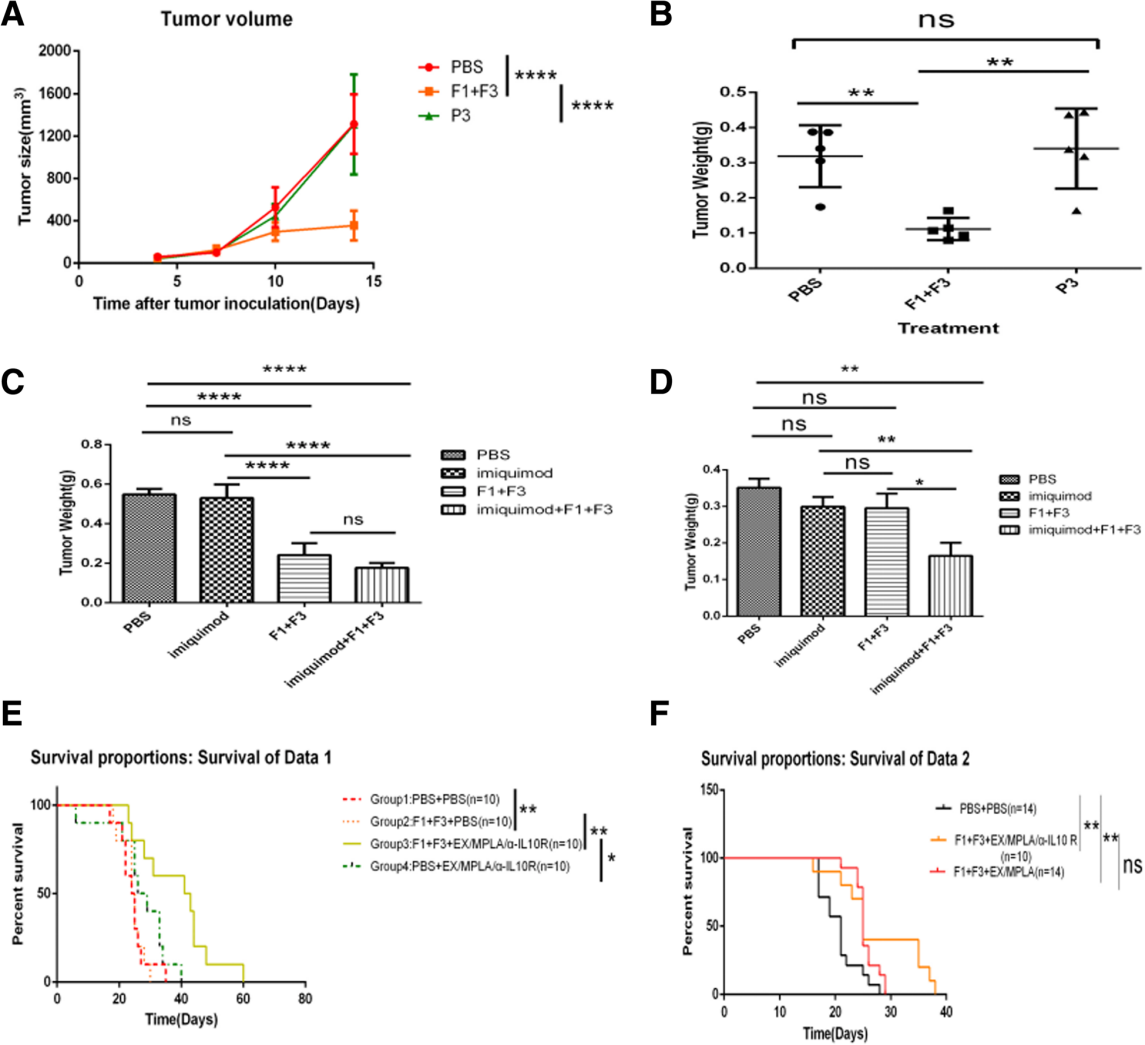
Caerin 1.1 and Caerin 1.9 inhibit TC-1 growth in vivo Groups of five C57/BL6 mice were subcutaneously transplanted with 2 × 10^5^ TC-1 tumour cells. When tumour size reached 3 mm, 30 μg of Caerin 1.1 (F1) and Caerin 1.9 (F3), or 30 μg of control peptide P3 was injected directly into tumour once per day for 7 consecutive days. Control group received PBS. Tumour growth were monitored every 3 days. Four days after final injection, tumours were isolated from individual mice and weighed. **a**: tumour growth curve; **b**: tumour weight. Groups of five C57/BL6 mice (**c**) or Nude mice (**d**) were subcutaneously transplanted with 2 × 10^5^ of TC-1 tumour cells. When tumour size reaches to 3 mm, 30 μg of Caerin 1.1 (F1) and Caerin 1.9 (F3), 50 μg of imiquimod, or same amounts of imiquimod and Caerin peptides were injected directly into tumour one time per day for consecutively 7 days. Control group received PBS. 4 days after final injection, tumours were isolated from individual mice and weighed. Results were pooled from two independent experiments. (**e**): Four groups of ten C57/BL6 mice were subcutaneously transplanted with 2 × 10^5^ of TC-1 tumour cells. 4 days after tumour inoculation, two groups were immunised twice 7 days apart with Ex/MPLA/anti-IL-10 receptor antibodies as described in Material and Methods. One day after the primary immunisation, mice were either injected for 7 consecutive days with 30 μg of caerin 1.1 and caerin 1.9 or PBS. The survival of tumour bearing mice were monitored. The results represent pooled data collected from two independent experiments. (**f**): Three groups of fourteen C57/BL6 mice were subcutaneously transplanted with 2 × 10^5^ of TC-1 tumour cells. Four days after tumour inoculation, two groups were immunized twice, 7 days apart, with Ex/MPLA/ Anti-IL-10 receptor antibody described above, or with Ex/MPLA only. Control group with PBS only. One day after the primary immunisation, mice were either injected for 7 consecutive days with 30 μg of caerin 1.1 and 1.9 or PBS. The survival of tumour bearing mice were monitored.

We then investigate whether caerin 1.1 and 1.9 were able to inhibit TC-1 cell growth in vivo. C57BL/6 mice were subcutaneously transplanted with TC-1 cells. Four days later, 30 μg of caerin 1.1 and 1.9, P3 or PBS was injected directly into the tumour daily for 7 consecutive days. Results showed that caerin peptides, but not P3 peptide or PBS was able to inhibit the TC-1 cell growth (Fig. 4a and b). We did not observe the weight loss after TC-1 tumour were treated with caerin peptides compared with untreated and P3 treated mice.

In similar experiment was conducted using intra-tumour injection of Caerin peptides, PBS, 50 μg of imiquimod, or imiquimod/peptides groups. Three days after the final injection, tumours were removed and weighed using an electronic scale. Caerin 1.1 and 1.9 significantly inhibited TC-1 tumour growth compared with PBS or Imiquimod only treated groups. At 50 μg per injection, Imiquimod was unable to inhibit TC-1 growth. However, caerin peptides combined with imiquimod treatment inhibited TC-1 tumour growth, although the inhibition was not statistically significant compared with caerin peptides treatment alone (Fig. 4c). When this experiment was repeated in nude mice, caerin 1.1 and 1.9 lost the ability of inhibiting tumour growth (Fig. 4d).

### Caerin 1.1 and 1.9 increase the efficacy of a HPV16 E7 peptide based therapeutic vaccine

We hypothesised that caerin peptides would increase the efficacy of a therapeutic vaccine, based on the observation that they had cytotoxicity on tumour cells and promoted the secretion of pro-inflammatory cytokines using quantitative proteomic analysis [20]. TC-1 tumour bearing mice were divided randomly into 4 groups: 1) Ex/MPLA/anti-IL-10R antibody immunisation and caerin 1.1/1.9 tumour local administration; 2) Ex/MPLA/anti-IL-10R antibody immunisation plus PBS tumour local injection; 3) caerin 1.1/1.9 tumour local injection and 4) PBS tumour local injection. TC-1 tumour bearing mice were immunised twice 7 days apart, followed by intra-tumour injection of caerin 1.1 and 1.9 or PBS. The result clearly showed that immunisation with EX/MPLA/anti-IL-10 antibody plus local administration of caerin peptides significantly increased the survival time of TC-1 tumour bearing mice. Immunisation with EX/MPLA/anti-IL-10R antibody alone, or tumour local injection of caerin peptides alone did not increase the survival time of TC-1 tumour bearing mice compared with PBS treated control group (Fig. 4e). Next, we compared whether caerin peptides local administration combined with EX/MPLA only immunisation also improved the survival time of TC-1 tumour bearing mice. The survival time of caerin treated TC-1 tumour bearing mice was significantly increased compared with PBS treated mice. Mice survived longer when immunised with EX/MPLA/anti-IL-10R antibody plus caerin 1.1/1.9 tumour local injection compared to the EX/MPLA only immunisation group plus caerin 1.1/1.9 local injection, however, there was no statistical difference (Fig. 4f). These results suggest that disturbing the immune suppressive microenvironment is critical for increasing the efficacy of a therapeutic vaccine.

### Caerin peptides treated TC-1 cells secrete more IL-6

**Fig. 5:**
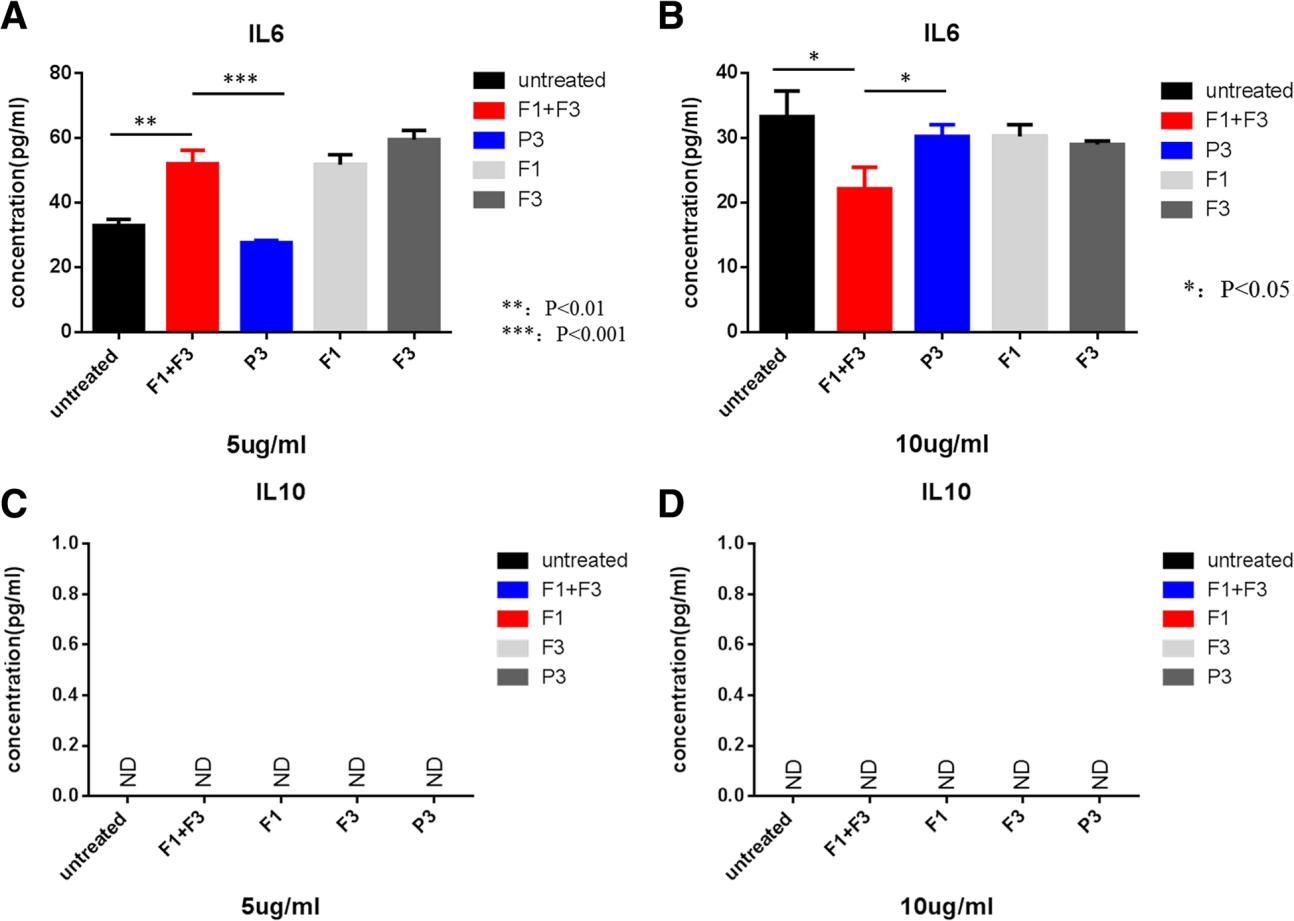
Caerin peptides induce TC-1 cells to secrete more IL-6 5 or 10 μg of Caerin 1.1 (F1) and Caerin 1.9 (F3) peptides or control peptide P3 were added to 8 × 10^3^ TC-1 cells in flat bottomed 96 well plates and cultured overnight at 37 °C with 5% CO_2_. IL-6 and IL-10 from the supernatants of caerin peptide treated TC-1 cells were detected by ELISA. IL-6 (**a**, **b**). IL-10 (**c**, **d**).

We investigated the possible mechanism of how caerin peptides might increase the efficacy of the therapeutic vaccine. Our proteomic analysis showed that caerin peptides could activate the NFκB signalling pathway of TC-1 cells and promote the secretion of pro-inflammatory cytokines such as IL-1β, IL-6, MCP-1 and MCP-3 [20]. Supernatant from caerin treated TC-1 cells was analysed by cytokine ELISA for IL-6 and IL-10. Caerin 1.1 and 1.9 at 5 μg/ml was able to stimulate TC-1 cells to secrete more IL-6. The IL-6 was increased to 55 pg/ml compared with 30 pg/ml of untreated TC-1 cells, while IL-10 was not detected (Fig. 5). At 10 μg/ml, IL-6 levels from caerin1.1/1.9 treated cells were lower than those from untreated cells, suggesting caerin 1.1/1.9 at this concentration might be toxic to the TC-1 cells. These results indicate that caerin peptides may not only have a cytotoxic effect on TC-1 cells, but also are able to stimulate TC-1 cells to secrete more inflammatory cytokines, stimulating a pro-inflammatory response.

### Caerin 1.1 and 1.9 attract more T cells to the tumour site

**Fig. 6:**
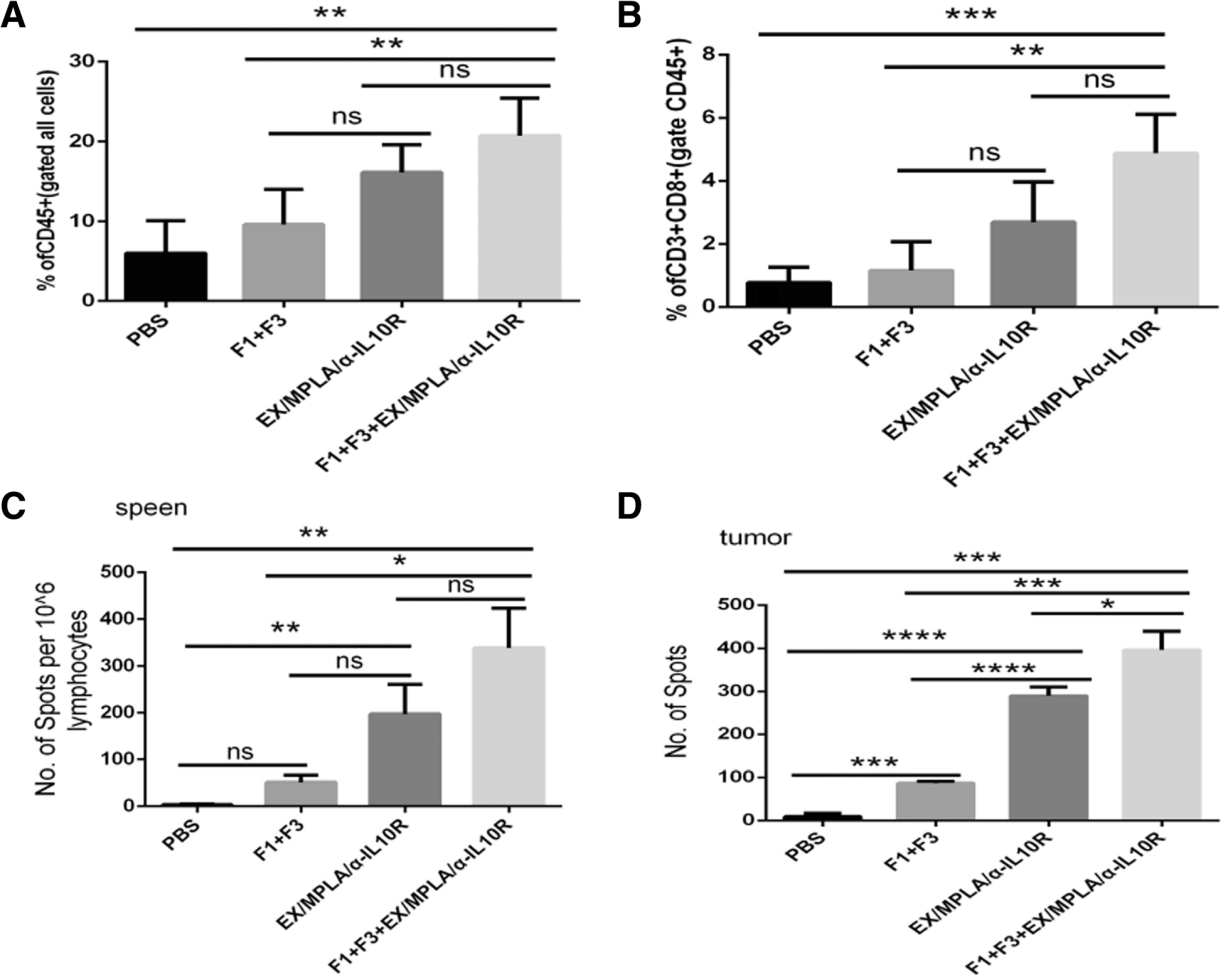
Caerin 1.1 and 1.9 attract more T cells to the tumour site Four groups of five C57/BL6 mice were subcutaneously transplanted with 2 × 10^5^ of TC-1 tumour cells. Four days after tumour inoculation, two groups were immunised twice 7 days apart with Ex/MPLA/anti-IL-10 receptor antibodies as described above. One day after the primary immunisation, mice were either injected for 7 consecutive days with 30 μg of caerin 1.1 and caerin 1.9, or PBS. When the tumor diameter was 1 cm in the control group, the mice were sacrificed and the tumor tissues were dissociated, digested and analysed by flow cytometry for surface molecules expression of CD45.2, CD4, CD8a and NK1.1. (**a**) Percentage of CD45.2 cells; (**b**) Percentage of CD3 + CD8+ cells gated on CD45.2 cells; (**c**) Percentage of CD3 + CD4+ cells gated on CD45.2 cells; (**d**) Percentage of CD3-NK1.1+ of CD45.2 cells in tumor tissue.

Next, we investigated whether caerin 1.1 and 1.9 attract more T cells to the tumour site. TC-1 tumour bearing mice were immunised twice with either Ex/MPLA/anti-IL-10 receptor antibody, or left unimmunised. This was followed by intra-tumour PBS or caerin 1.1 and 1.9 treatment (Fig. 6). Seven days after final immunisation, tumour infiltrating T cells were analysed by either flow cytometry or Elispot assay. Local administration of caerin peptides 1.1 and 1.9 to the tumour bearing mice vaccinated with Ex/MPLA/anti-IL-10 receptor antibody attracted more CD45+ and CD3+ cell to tumour site but this was not statistically significant compared with vaccination without caerin 1.1 and 1.9 treatment in the flow cytometry analysis (Fig. 6). However, if HPV16 E7 antigen specific CD8+ T cells were examined by Elispot assay, local administration of caerin1.1/1.9 peptides plus immunisation resulted in significantly higher antigen specific CD8+ T cells infiltrating to the tumour site, compared with immunisation alone. Splenic E7 specific CD8+ T cells in caerin1.1/1.9 treated and vaccinated group, compared with immunisation group alone, were higher but without statistical significance (Fig. 6).

## Discussion

Therapeutic vaccines are efficient against HPV infection related pre-cancer conditions, such as VIN3 and CIN3 [9], but remain to show efficacy against cervical cancer [8]. The low immunogenicity of therapeutic vaccines and the tumour immune suppressive microenvironment are key factors that limit the efficacy of a cancer therapeutic vaccine [1, 14, 17, 19]. To be effective, a therapeutic vaccine should elicit high quality, and sufficient numbers of effector T cells that are able to migrate to the tumour site, overcome the immune suppressive environment and kill tumour or viral infected cells [7].

In the current study, we demonstrated that immunisation with four overlapping peptides covering entire HPV16 E7 protein (EX) with MPLA as adjuvant elicits significantly greater numbers of E7 specific CD8+ T cells when IL-10 signalling is blocked. However, the EX/MPLA/anti- IL-10 receptor antibody immunisation did not prolong the survival time of TC-1 tumour bearing mice compared with the same vaccine immunisation without IL-10 signalling blockade (Fig. 1). Increased inhibition of TC-1 tumour growth and survival of tumour bearing mice were only observed when tumour challenge was performed after EX/MPLA/anti-IL10R antibody vaccination (Fig. 1). Tumour infiltrating T cells isolated 3 weeks after TC-1 tumour transplantation, secreted less IFNγ, perforin and Granzyme B compared with those isolated 2 weeks after TC-1 tumour inoculation (Fig. 2). We also demonstrated that caerin peptides isolated from the skin secretion of Australian tree frog are able to promote the secretion of pro-inflammatory IL-6 by TC-1 cells (Fig. 5). Moreover, our studies showed the caerin peptides were i) functionally stable (Fig. 4); ii) were able to inhibit TC-1 tumour growth in vivo; iii) that the inhibition require adaptive immune responses (Fig. 4); and iv) increased the efficacy of the Ex/MPLA/anti-IL-10R vaccine (Fig. 4). Finally, we demonstrated a mechanism by which caerin peptides use to enhance the efficacy of the therapeutic vaccine by recruiting more T cells to the tumour site (Fig. 6).

Vaccine induced CD8 + T cells can predict the therapeutic efficacy of a vaccine against the tumour [12]. Great efforts have been made to increase the immunogenicity of therapeutic vaccines. We and others have previously demonstrated that blocking IL-10 at the time of immunisation drastically increase the vaccine induced CD8+ T cell response [14, 25, 26]. Studies also showed that this immunisation strategy better prevents tumour growth than the immunisation without IL-10 signalling blockade, if IL-10 signalling was blocked by *i.p.* injection of anti-IL-10 receptor antibodies [25]. Subcutaneous administration of vaccine and anti-IL-10 receptor antibodies increases the vaccine induced CD8 T cell responses compared with immunisation without IL-10 signalling blockade, but is not as significant as administration of anti-IL-10 through *i.p*. injection [16]. In the current study, however, HPV16 E7 peptide-based vaccine was unable to prolong the survival time of TC-1 tumour bearing mice when IL-10 signalling was blocked through subcutaneously administration of anti-IL-10 receptor antibodies at the time of immunisation (Fig. 1). This outcome is similar to most clinical trial results in that therapeutic vaccines are yet to show any clinical efficacy against cervical cancer [1, 27, 28].

Cancer develops after lengthy battle with the human immune system. The tumour microenvironment not only promotes tumour development and metastasis, but also prevents tumour killing by effector cells of the adaptive immune system, mostly T cells [17]. T cells eventually lose their function during chronic viral infection and tumour development [29]. The centre of a solid tumour usually lacks of oxygen, and hypoxia of the solid tumour inhibits CD4+ T cell function [30]. Our results also showed that tumour infiltrating CD4+ and CD8+ T cells isolated 3 weeks after TC-1 tumour inoculation secrete less IFN*γ* compared with those isolated at 3 weeks, whether or not the T cells express PD-1. Tumour infiltrating CD8+ T cells isolated at 3 weeks also express less Granzyme B and perforin (Fig. 2). These results agree with clinical trial results [1] in that therapeutic vaccines are not effective against cervical cancer, arguing that when the tumour microenvironment is established, the vaccine-induced T cells will not kill the tumour cells efficiently.

Targeting the tumour microenvironment has recently shown exciting outcomes, from the use of antibody targeting immune-checkpoints, such as anti-PD-1 [31]. Therapeutic vaccines combined with anti-PD-1 enhance the efficacy of the therapeutic vaccines against different solid tumours, both in animal models and clinical trials [32, 33]. Recently, we demonstrated that naturally derived peptides isolated from Australian tree frog are able to inhibit multiple tumour growth in vitro, partially through inducing apoptosis of tumour cells, by penetrating the tumour cell membrane into the cytosol [20]. Proteomic analysis showed that caerin peptides activate multiple cell signalling pathways including NFκB to stimulate pro-inflammatory cytokines and chemokines. These results prompted us to investigate whether caerin peptides can be used to target the tumour microenvironment. Indeed, caerin peptides are able to inhibit both TC-1 in vitro and promote TC-1 cells to secrete more IL-6 (Fig. 5). Caerin peptides are environmentally stable, as their biological activities remaining identical when stored at room temperature, in acidic condition and at high temperatures (100 °C) (Fig. 3), suggesting these peptides are ideal for in vivo administration. Indeed, when combining tumour local administration of caerin peptides with Ex/MPLA vaccine to TC-1 tumour bearing mice, the survival time was significantly extended compared with vaccination alone or caerin peptide treatment alone, whether IL-10 signalling is blocked or not.

Caerin peptides mediated increased efficacy of therapeutic vaccine via recruiting more T cells to the tumour site. After administration of caerin peptide 1.1/1.9, T cell numbers as well as antigen specific CD8+ T cells migrating to TC-1 tumour are significantly increased compared with untreated mice. Moreover, the number of antigen specific CD8+ T cells migrating to TC-1 tumour is significantly increased in vaccine plus caerin treatment group compared with vaccine only group. It is likely that caerin peptides increase the levels of chemokines, which attract T cells to the tumour site.

The efficacy of caerin peptides targeting the tumour microenvironment can be further improved. Previously, we demonstrated that nanomaterial graphene oxide can incorporate IFN*γ* and anti-IL-10 receptor antibodies [34]. The graphene oxide absorbed antibodies and cytokine retained their biological activities both in vivo and in vitro. They are slowly released to the outside environment compared with free cytokines and antibodies [34]. Graphene oxide absorbed anti-IL10R antibodies are more efficient than free anti-IL10R antibodies at eliciting LPS stimulated CD8 T cell responses. Recently, radioactive iodine 125 (^125^I) was labelled to the caerin 1.9 peptide. The ^125^I labelled caerin 1.9 was almost 100 time more efficient at inhibiting tumour growth in vitro compared with non-labelled caerin 1.9, more efficient than free ^125^ I [23]. Caerin peptides may therefore be used to disturb the tumour microenvironment if properly incorporated in a controlled release reagent, to increase the efficacy of cancer therapeutic vaccines against cancers.

## Conclusion

Taken together, caerin peptides are able to boost the efficacy of a HPV16 E7 peptide-based vaccine containing IL-10 signalling inhibitor to better inhibit the HPV16 E6/E7 transformed TC-1 growth and extend the survival time of TC-1 tumour bearing mice. Mechanisms involved include disturbing the tumour microenvironment by recruiting more antigen specific T cells to the tumour site. These results warrant ongoing investigations in a clinical setting.

## Data Availability

All data is included in this manuscript. Any possibly related data and materials are available on request.

## Abbreviations

Caerin 1.1: ^1^GLLSVLGSV^10^AKHVLPHVLP^20^HVVPVIAEHL-NH2
caerin 1.9: ^1^GLFGVLGSI^10^AKHVLPHVVP^20^VIAEKL-NH2
HPV: Human papillomavirus
IDO: Indoleamine-pyrrole 2, 3-dioxygenase
IL-10: Interleukin 10
Mab: monoclonal antibody
MPLA: monophosphoryl lipid A
VLPs: Virus like particles
